# Non-pharmacological interventions of travel restrictions and cancelation of public events had a major reductive mortality affect during pre-vaccination coronavirus disease 2019 period

**DOI:** 10.3389/fmed.2022.914732

**Published:** 2022-08-22

**Authors:** Haoyu Wen, Fang Shi, Yan Liu, Cong Xie, Guiyu Qin, Fang Wang, Xiaoxue Liu, Jianjun Bai, Qiumian Hong, Runxue Ma, Chuanhua Yu

**Affiliations:** ^1^Department of Epidemiology and Biostatistics, School of Public Health, Wuhan University, Wuhan, China; ^2^Hubei Provincial Center for Disease Control and Prevention, Institute of Preventive Medicine Information, Wuhan, China; ^3^Department of Biostatistics, School of Public Health, Xuzhou Medical University, Xuzhou, China; ^4^China Global Health Institute, Wuhan University, Wuhan, China

**Keywords:** COVID-19, vaccines, public health interventions, random forest, mortality

## Abstract

**Background:**

The coronavirus disease 2019 (COVID-19) is a severe acute respiratory disease that poses a continuous threat to global public health. Many non-pharmacological interventions (NPIs) have been implemented to control the COVID-19 pandemic since the beginning. The aim of this study was to assess the impact of various NPIs on COVID-19 mortality during pre-vaccination and vaccination periods.

**Methods:**

The COVID-19 data used in this study comes from Our World in Data, we used the Oxford Strict Index (OSI) and its five combination interventions as independent variables. The COVID-19 mortality date (MRT) was defined as a date when daily rate of 0.02 COVID-19 deaths per 100,000 population in a country was reached, and the COVID-19 vaccination date (VRT) was defined as people vaccinated reaching 70%. Linear regression and random forest models were used to estimate the impact of various NPI implementation interventions during pre-vaccination and vaccination periods. The performance of models was assessed among others with Shapley Additive Explanations (SHAP) explaining the prediction capability of the model.

**Results:**

During the pre-vaccination period, the various NPIs had strong protective effect. When the COVID-19 MRT was reached, for every unit increase in OSI, the cumulative mortality as of June 30, 2020 decreased by 0.71 deaths per 100,000 people. Restrictions in travel (SHAP 1.68) and cancelation of public events and gatherings (1.37) had major reducing effect on COVID-19 mortality, while staying at home (0.26) and school and workplace closure (0.26) had less effect. Post vaccination period, the effects of NPI reduced significantly: cancelation of public events and gatherings (0.25), staying at home (0.22), restrictions in travel (0.14), and school and workplace closure (0.06).

**Conclusion:**

Continued efforts are still needed to promote vaccination to build sufficient immunity to COVID-19 in the population. Until herd immunity is achieved, NPI is still important for COVID-19 prevention and control. At the beginning of the COVID-19 pandemic, the stringency of NPI implementation had a significant negative association with COVID-19 mortality; however, this association was no longer significant after the vaccination rate reached 70%. As vaccination progresses, “cancelation of public events and gatherings” become more important for COVID-19 mortality.

## Introduction

Corona virus disease 2019 (COVID-19) is a highly concealed and highly transmissible severe acute respiratory disease caused by the severe acute respiratory syndrome coronavirus 2 (SARS-CoV-2) (1). The World Health Organization (WHO) announced that COVID-19 has developed into a “pandemic” on March 11, 2020 (2). COVID-19 is a continuous threat to global public health. According to WHO statistics, as of December 31, 2021, a total of 285,581,643 COVID-19 cases and 5,428,033 deaths from COVID-19 have been reported worldwide (3). The number of COVID-19 cases and deaths continues to grow rapidly.

Before the COVID-19 vaccine was invented and widely used, various non-pharmacological interventions (NPIs) had been implemented in most countries around the world to cope with the sharp increase in the COVID-19 cases and deaths and to maintain the normal operation of the healthcare system. In Wuhan, China, a series of multifaceted interventions resulted in significant mitigation of the COVID-19 outbreak (4). Italy was the first European country to carry out interventions to deal with COVID-19, and other countries followed suit (5). The interventions, largely successful in curbing the spread of COVID-19, incurred economic and social costs, including increased unemployment (6), declined income, education interruption, social isolation and related socio-psychological consequences (7). Gaining a better understanding of when and how these interventions can effectively control COVID-19 is critical for health prevention and control experts to implement a specific sequence of key countermeasures judiciously and timely.

On November 18, 2020, Pfizer/BioNTech became the first in the world to release full late-stage trial data for the COVID-19 vaccine. Shortly after, on December 8, 2020, the United Kingdom became the first of all countries to vaccinate the public with COVID-19 (8). According to the WHO, as of December 31, 2021, the countries with largest proportion of population vaccinated are Gibraltar, Pitcairn Islands, United Arab Emirates, all with a proportion of more than 90% (3). In this case, an important issue is how should we better implement NPI as vaccination progresses? Previous studies have focused on the early stages of the COVID-19 outbreak, exploring the association between NPI and COVID-19 mortality (9–14). However, there is a lack of research on changes in this association after vaccination, and a lack of exploration of the effectiveness of NPI after vaccination as well.

Hence, to address these limitations, this study aimed to assess the impact of various NPIs on COVID-19 mortality during pre-vaccination and vaccination periods. This study uses the linear regression to find out the association between NPI and COVID-19 mortality, and investigate the priority of NPI by random forest model.

## Materials and methods

### Data source

The data used in this study comes from Our World in Data (OWID) (15). OWID provides statistics on the coronavirus pandemic in 207 countries/regions around the world. Data on COVID-19 deaths comes from the European Center for Disease Control and Prevention and Johns Hopkins University, and the vaccination dataset is the most recent official numbers from governments and health ministry’s worldwide (16). The population estimates for per capita indicators are based on the United Nations World Population Prospects (17).

### Methods

In this study, the COVID-19 mortality rate was the outcome variable, the Oxford Strict Index (OSI) and the interventions included were independent variables. The reason why this study chose COVID-19 mortality rather than COVID-19 incidence as the outcome variable is that the former is more reliable. The incidence data of COVID-19 depends largely on the testing capacity, which could cause great data inaccuracy (18). The Oxford Stringency Index (19) records the strictness of the intervention that primarily restricts people’s behavior, which is a composite index based on nine interventions: school closures, workplace closures, cancelation of public events, restrictions on public gatherings, closures of public transport, stay-at-home requirements, public information campaigns, restrictions on internal movements, international travel controls (the definitions of these nine NPIs are provided in Supplementary Table 1). According to the classification principles of OWID, we further transformed these nine interventions into five intervention combinations: “school and workplace closures,” “cancelation of public events and gatherings,” “stay-at-home restrictions,” “public information campaigns,” “restrictions in international and domestic travel.” NPI combination “school and workplace closures” contains NPI “schools closures” and “workplaces closures”; NPI combination “cancelation of public events and gatherings” contains “cancelation of public events” and “restrictions on public gatherings”; NPI combination “restrictions in international and domestic travel” contains “closures of public transport,” “restrictions on internal movement,” “international travel controls.” The stringency of an NPI combination is calculated as the average of the stringency of the NPIs it contains.

In this study, we set two thresholds: the COVID-19 mortality rate threshold (MRT) and the COVID-19 vaccination rate threshold (VRT). The COVID-19 MRT is defined as a daily rate of 0.02 new COVID-19 deaths per 100,000 people (based on a 7-day moving average), and the COVID-19 VRT is defined as people vaccinated (one dose or two doses) per hundred reaching 70%. Since vaccination is ongoing, the number of countries reaching the COVID-19 VRT is increasing. In order to maintain the certainty of the countries chosen in this study, we only selected countries reaching the COVID-19 VRT (the proportion of population vaccinated greater than 70%) on or before October 31, 2021. Finally, based on the above two thresholds, 34 countries with more than 250,000 inhabitants and for which relevant data were available were included (Specific country names are shown in Supplementary Documents).

### Linear regression

In this study, we established two linear regression models, “Lm1” and “Lm2.” “Lm1” used the OSI on the day a country reached the COVID-19 MRT as the independent variable, and used the cumulative COVID-19 death rate per 100,000 people on June 30, 2020 as the dependent variable; “Lm2” used the OSI on the day a country reached the COVID-19 VRT as the independent variable, and the cumulative death rate per 100,000 people between the day the COVID-19 VRT was reached and December 31, 2021 was used as the dependent variable. June 30, 2020 was chosen in “Lm1” because on that day the new COVID-19 death rate fell to relatively low level in almost all 34 countries; and December 31, 2021 was chosen in “Lm2” as the data was the latest available to date.

In addition, the regression models “Lm1” and “Lm2” control for the same 11 health-related indicators as covariates: the date the threshold was reached, because the effect of NPI is closely related to time; the number of hospital beds per 1,000 people is taken as a measure of baseline health care capacity; proportion of people aged 65 older, because age is an important risk factor for COVID-19 death; female smoking prevalence, male smoking prevalence and diabetes prevalence reflect the basic health status of the population, population density, because higher population density leads to higher exposure rates; per capita GDP and the share of people living in extreme poverty to explain the wealth difference; the human development index and life expectancy reflect the comprehensive health level of a country. Health-related covariate data for the 34 countries included in the study are presented in Supplementary Table 2.

### Random forest

The decision tree model is a tree structure composed of root nodes, branch nodes and leaf nodes, reflecting the mapping relationship between features and tags. Random forest (RF) is an ensemble learning method based on decision trees (20). The RF model can be briefly understood as the following 4 steps (21): (1) randomly select k samples from the given dataset (k is usually equal to 2/3 of the dataset) for training the model, and the remaining samples are used to estimate the RF’s goodness of fit; (2) from each sample with m variables, randomly select a subset with n variables (n < m) and create a decision tree; (3) each tree grows at a constant n over a maximum extent, without pruning, until it cannot split.; (4) calculate the prediction result for each tree, and the average prediction of all trees is used to create the final output.

The RF model in this study is generated based on 500 decision trees. We use 70% of the dataset as the training set and the remaining 30% as the test set. RMSE (root mean square error), MAE (mean absolute error), MSE (mean square error), and MAPE (mean absolute percentage error) were used to assess the performance of the random forest model. In this study, we generated two random forest regression models: “RF1”and “RF2.” “RF1” used the stringency of the five NPI combinations on the day a country reached the COVID-19 MRT as the independent variable and uses the same dependent variable as “Lm1”; “RF2” used the stringency of the five NPI combinations on the day a country reached the COVID-19 VRT as the independent variable and uses the same dependent variable as “Lm2.” These two random forest models included the same covariates as in the previous linear regression.

We ranked NPIs by three importance measures: (a) permutation based feature importance, (b) Gini-based importance, and (c) feature importance computed with Shapley Additive Explanations (SHAP) Values. The permutation based feature importance is measured using mean decrease in accuracy (MDA). MDA is a method of computing the feature importance on permuted out-of-bag (OOB) samples based on mean decrease in the accuracy (22). The Gini-based importance is measured using mean decrease in Gini (MDG). MDG is a measure of the contribution of individual variables to the homogeneity of the nodes in a random forest model. Each node split is compared to the original model Gini coefficient, which is a measure of the statistical dispersion of node homogeneity across all runs (23). The changes in Gini are summed for each variable and normalized, variables with higher node purity have a higher decrease in Gini coefficient. And for feature importance computed with SHAP Values, which were based on “Shapley values” developed by Shapley in the cooperative game theory (24). The goal of SHAP is to explain the prediction of an instance x by computing the contribution of each feature to the prediction. The SHAP explanation method computes Shapley values from coalitional game theory, where the feature values of a data instance act as players in a coalition (25). We also explored the correlation of the NPIs importance rankings obtained by the three importance measures through the Spearman correlation coefficient.

## Results

### Linear regression

**FIGURE 1:**
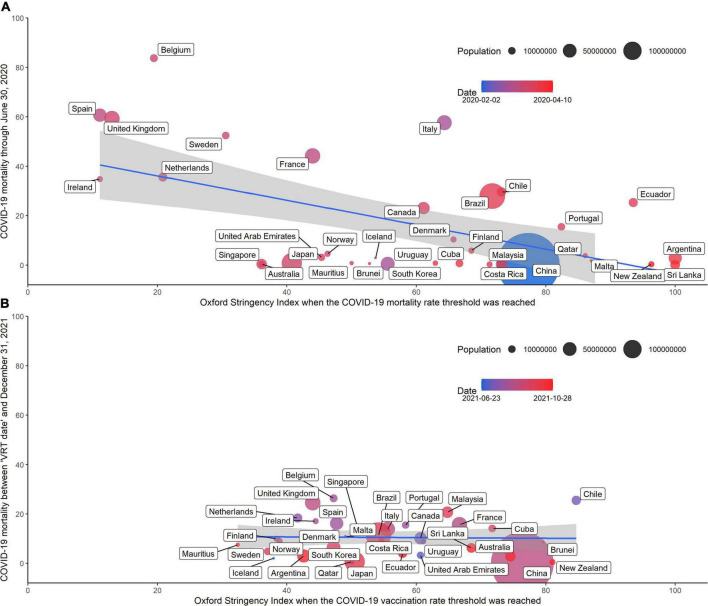
**(A)** The OSI on the day of reaching the COVID-19 mortality rate threshold and cumulative COVID-19 mortality through June 30, 2020. **(B)** The OSI on the day of reaching the COVID-19 vaccination rate threshold and cumulative COVID-19 mortality between the day of reaching the COVID-19 vaccination rate threshold and December 31, 2021.

Among the 34 countries included in the study, the date of reaching the COVID-19 MRT ranges from February 2, 2020 in China to April 10, 2020 in New Zealand, and the OSI on the date of reaching the COVID-19 MRT ranges from 11.11 in Spain and Iceland to 100 in Argentina and Sri Lanka (Supplementary Table 3). Countries with higher OSI when reaching COVID-19 MRT have a lower cumulative COVID-19 mortality on June 30, 2020 (Figure 1). This association persisted after controlling for the aforementioned 11 health-related covariates (Supplementary Table 4). When the COVID-19 MRT is reached, for every unit increase in OSI, the cumulative mortality rate as of June 30, 2020 will decrease by 0.71 deaths per 100,000 people (95% CI = −1.08 to −0.34 per 100,000 people).

Among the 34 countries included in the study, the date the country reached the COVID-19 VRT ranges from June 23, 2021 in Iceland to October 28, 2021 in Ecuador, and the OSI on the date of reaching the COVID-19 VRT ranges from 32.41 in Mauritius to 84.72 in Chile. However, the OSI on the day of reaching the COVID-19 VRT and the cumulative COVID-19 death rate per 100,000 people between the day the COVID-19 VRT was reached and December 31, 2021 did not show a significant association (Figure 1). After adjusting for the aforementioned covariates, the *P*-value of the “Lm2” is greater than the significance level (α = 0.05).

### Random forest model

**FIGURE 2:**
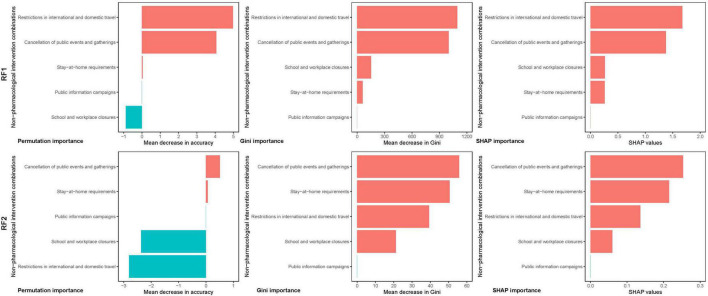
Ranking of the importance of NPI combinations to COVID-19 mortality.

The most common NPI combinations these countries implemented when they reached the COVID-19 MRT was “public information campaigns” (33 out of 34 countries), while the NPI combinations implemented by the fewest countries was “stay-at-home requirements” (20 out of 34 countries) (see Table 1). For a country, in addition to the number of NPIs implemented, the strictness of the implementation of NPIs is also important. Among the 34 countries included in this study, NPI combinations “public information campaigns” (33/34) was implemented with the strictest standards by the most countries, and followed by “school and workplace closures” (10/34) and “cancelation of public events and gatherings” (10/34). According to the RF model, the most important NPI combination for COVID-19 mortality is “restrictions in international and domestic travel” and “cancelation of public events and gatherings” (see in Figure 2). All three importance measures indicated that “restrictions in international and domestic travel” (SHAP 1.68) and “cancelation of public events and gatherings” (1.37) had major reducing effect on COVID-19 mortality, while “stay-at-home requirements” (0.26) and “school and workplace closure” (0.26) had less effect. Based on MAE, MSE, RMSE, MAPE, we can see that the random forest model performs well (see in Supplementary Table 5).

**TABLE 1:** Implementation of non-pharmacological intervention combinations at the date the COVID-19 mortality rate threshold was reached in 34 countries.

Location	MRT date	School and workplace closures	Cancelation of public events and gatherings	Restrictions in international and domestic travel	Stay-at-home requirements	Public information campaigns
Argentina	2020/3/8	2.0	2.5	1.0	0.0	2.0
Australia	2020/3/1	2.5	3.0	2.3	2.0	2.0
Belgium	2020/3/11	2.0	1.0	1.0	1.0	2.0
Brazil	2020/3/20	0.0	2.0	1.0	0.0	2.0
Brunei	2020/3/28	2.5	2.5	1.7	2.0	2.0
Canada	2020/3/9	2.0	1.0	2.0	1.0	2.0
Chile	2020/3/22	3.0	2.5	1.3	1.0	2.0
China	2020/1/28	0.0	1.5	0.0	0.0	2.0
Costa Rica	2020/3/19	2.0	2.0	2.0	1.0	2.0
Cuba	2020/3/18	2.5	3.0	2.7	1.0	2.0
Denmark	2020/3/14	3.0	3.0	1.7	2.0	2.0
Finland	2020/3/21	0.0	0.5	0.3	0.0	2.0
France	2020/3/5	0.0	0.0	0.0	0.0	2.0
Iceland	2020/3/21	0.0	0.0	0.0	0.0	2.0
Ireland	2020/3/11	1.5	3.0	0.3	0.0	2.0
Israel	2020/3/20	2.0	3.0	1.7	1.0	2.0
Italy	2020/2/24	2.5	1.0	1.0	0.0	2.0
Japan	2020/3/10	0.0	0.0	0.0	1.0	2.0
Malaysia	2020/3/17	3.0	3.0	1.3	3.0	2.0
Malta	2020/4/8	3.0	3.0	1.3	2.0	2.0
Mauritius	2020/3/21	2.0	2.0	1.3	1.0	2.0
Netherlands	2020/3/6	1.5	1.0	2.3	2.0	2.0
New Zealand	2020/3/29	3.0	2.0	1.7	1.0	2.0
Norway	2020/3/14	0.0	1.5	1.0	0.0	2.0
Portugal	2020/3/17	3.0	2.5	2.3	1.0	2.0
Qatar	2020/3/28	2.0	0.5	1.0	0.0	2.0
Singapore	2020/3/21	1.5	2.5	1.3	0.0	2.0
South Korea	2020/2/23	0.0	2.0	1.0	0.0	2.0
Spain	2020/3/3	2.5	1.0	2.7	0.0	2.0
Sri Lanka	2020/3/28	3.0	3.0	2.7	2.0	2.0
Sweden	2020/3/10	3.0	3.0	2.7	3.0	2.0
United Arab Emirates	2020/3/20	3.0	1.0	1.7	0.0	0.0
United Kingdom	2020/3/10	3.0	3.0	2.7	3.0	2.0
Uruguay	2020/3/28	3.0	2.5	2.7	2.0	2.0

The numbers in the table represent the strictness of NPI combination implementation. The stringency of an NPI combination is calculated as the average of the stringency of the NPIs it contains. For the specific meaning of the strictness of the single NPI, see in Supplementary Table 1. MRT date, the date the COVID-19 mortality rate threshold was reached.

For countries reaching the COVID-19 VRT, the most common implemented NPI combinations were “public information campaigns,” “cancelation of public events and gatherings” and “restrictions in international and domestic travel” (34 out of 34 countries); while the NPI combination with the fewest implementing countries is “stay-at-home requirements” (22 of 34) (see in Table 2). The NPI combination “public information campaigns” with strictest standard were still enforced in the most countries (34/34), followed by “cancelation of public events and gatherings” (12/34). When reaching the COVID-19 VRT, all three importance measures indicated that “cancelation of public events and gatherings” had the greatest impact on COVID-19 mortality, followed by “stay-at-home requirements.” However, after reaching the COVID-19 VRT, the effects of all NPIs on COVID-19 mortality were significantly lower than before: “cancelation of public events and gatherings” (SHAP 0.25), “stay-at-home requirements” (0.22), “restrictions in international and domestic travel” (0.14) and “school and workplace closure” (0.06). The Gini-based importance ranking and SHAP importance ranking hold strong correlations, and the correlation was significant (*p* < 0.05). Permutation based importance correlates weakly with both above (see in Supplementary Tables 6, 7).

**TABLE 2:** Implementation of non-pharmacological intervention combinations at the date the COVID-19 vaccination rate threshold was reached in 34 countries.

Location	VRT date	School and workplace closures	Cancelation of public events and gatherings	Restrictions in international and domestic travel	Stay-at-home requirements	Public information campaigns
Argentina	2021/10/21	1.0	1.0	1.3	1.0	2.0
Australia	2021/10/14	2.5	3.0	2.3	2.0	2.0
Belgium	2021/8/4	1.5	2.5	1.0	0.0	2.0
Brazil	2021/9/27	2.0	1.5	2.0	2.0	2.0
Brunei	2021/10/4	3.0	3.0	1.7	2.0	2.0
Canada	2021/7/20	2.0	2.5	2.3	1.0	2.0
Chile	2021/7/15	3.0	3.0	2.7	3.0	2.0
China	2021/8/26	3.0	3.0	1.7	3.0	2.0
Costa Rica	2021/10/18	2.0	2.0	0.7	1.0	2.0
Cuba	2021/9/18	2.5	3.0	2.0	2.0	2.0
Denmark	2021/7/24	1.5	2.0	1.0	0.0	2.0
Finland	2021/8/19	1.5	2.5	1.7	1.0	2.0
France	2021/8/20	1.0	2.5	0.7	1.0	2.0
Iceland	2021/6/23	1.5	2.5	2.0	0.0	2.0
Ireland	2021/8/12	1.0	1.5	1.0	0.0	2.0
Israel	2021/10/4	1.5	2.0	1.0	0.0	2.0
Italy	2021/8/28	1.5	1.5	2.0	2.0	2.0
Japan	2021/9/29	1.5	1.5	1.7	1.0	2.0
Malaysia	2021/9/24	2.5	0.5	2.0	2.0	2.0
Malta	2021/7/1	1.0	3.0	1.0	0.0	2.0
Mauritius	2021/10/21	1.0	1.5	0.7	0.0	2.0
Netherlands	2021/7/25	1.5	0.5	1.3	0.0	2.0
New Zealand	2021/10/7	3.0	3.0	2.7	2.0	2.0
Norway	2021/8/25	1.0	0.5	1.0	1.0	2.0
Portugal	2021/8/2	1.5	3.0	1.3	0.0	2.0
Qatar	2021/7/31	1.0	2.5	1.0	1.0	2.0
Singapore	2021/7/17	1.5	2.5	1.0	1.0	2.0
South Korea	2021/9/17	2.0	3.0	0.7	0.0	2.0
Spain	2021/8/4	1.0	3.0	0.7	2.0	2.0
Sri Lanka	2021/10/26	0.5	3.0	2.0	1.0	2.0
Sweden	2021/9/26	0.0	2.0	1.0	1.0	2.0
United Arab Emirates	2021/7/5	1.5	3.0	2.0	0.0	2.0
United Kingdom	2021/8/24	1.5	2.5	1.3	0.0	2.0
Uruguay	2021/7/15	2.0	2.0	2.0	1.0	2.0

The numbers in the table represent the strictness of NPI combination implementation. The stringency of an NPI combination is calculated as the average of the stringency of the NPIs it contains. For the specific meaning of the strictness of the single NPI, see in Supplementary Table 1. VRT date, the date the COVID-19 vaccination rate threshold was reached.

## Discussion

This study found that the stringency of NPI implementation was strongly negatively associated with COVID-19 mortality in the early stage of COVID-19 pandemic, and this association was no longer significant after COVID-19 vaccination rate reached 70%. As vaccination progressed, the most important NPI combinations changed from “restrictions in international and domestic travel,” “cancelation of public events and gatherings” to “cancelation of public events and gatherings.”

Since the emergence of COVID-19 at the end of 2019, it has been raging around the world for about 2.5 years. Until COVID-19 vaccines were invented, NPIs were the most effective way for countries to fight against COVID-19. Large-scale social distance intervention saved time for health services to treat cases and increase treatment capacity. Many studies have proved the effectiveness of interventions (9–11), however, the implementation of many interventions came with great social and economic costs. For example, the closure of educational facilities would interrupt learning and could lead to malnutrition, stress, and social isolation among children (26–28). The intervention “stay-at-home requirements” has significantly increased the incidence of domestic violence in many countries, with a huge impact on women and children (27). It also limits access to long-term care (such as chemotherapy), with a substantial impact on the health and survival chances of patients, especially for critically ill patients (29, 30). Therefore, the government must strike an acceptable balance between benefits and drawbacks when implementing interventions.

The ultimate goal of COVID-19 prevention and control is to reduce the mortality rate of COVID-19 to an extremely low and acceptable level, and to turn the epidemic into a more benign, endemic and cold-causing disease, on the premise that people are not restricted by large-scale interventions (31). Vaccination is considered the most likely way to achieve this. Existing studies have shown that most currently used COVID-19 vaccines are highly effective (>90%) against SARS-CoV-2 infection, symbolic COVID-19 disease, severe COVID-19 disease, and COVID-19 death at 2 months or less after vaccination (32–34). The negative association between OSI and COVID-19 mortality was not significant under COVID-19 VRT, which does not mean that NPI is no longer important for COVID-19 prevention and control at 70% COVID-19 vaccination rate, nor does it mean that a 70% vaccination rate is equivalent to the herd immunity threshold. Early relaxation of NPIs, before sufficient immunity has been established, could trigger a wave of infections that lead to hospitalizations and deaths (35). To build adequate immunity in the population, we need to consider not only about vaccinating the general population, but also about vaccinating the most vulnerable populations who need protection against disease. The lack of vaccination in highly susceptible pockets in the population could trigger small outbreaks and reduce the effect of population immunity (35). In addition, it is crucial to understand the drivers of vaccine hesitancy (36–38) and solve the inequality of vaccination opportunities (39).

On longer timescales, the possibilities of waning immunity and SARS-CoV-2 variants could lead to reduced immunity in the population. Six months after vaccination, the effectiveness of the COVID-19 vaccine against SARS-CoV-2 infection, symbolic COVID-19 disease decreased by more than 20 percentage points, but the effectiveness against severe COVID-19 disease and COVID-19 death waned limited, still around 80% (32–34, 40). Given that preventing of severe disease and death remains the primary goal of COVID-19 vaccination, this limited decline in vaccine efficacy or effectiveness for severe disease and death is acceptable. A seasonal vaccination program against SARS-CoV-2 similar to seasonal influenza vaccinations may be implemented in the future to counteract declining immunity (41). In addition, the booster dose of COVID-19 vaccine is also considered a way to combat declining immunity (32, 42). The SARS-CoV-2 variants are rapidly developing, currently including Alpha variant (B.1.1.7), Beta variant (B.1.351), Gamma variant (P.1), Delta variant (B.1.617.2), Omicron variant (B.1.1.529, BA.1, BA.1.1, BA.2, BA.3, BA.4, and BA.5) and so on (43). Compared with the previous variants, the Delta variant is more than twice as contagious, and may cause more severe illness in unvaccinated people (44–46). And the Delta and Omicron variants may be immune escape, leading a breakthrough infection of COVID-19 (47, 48). The emergence of the variants of SARS-CoV-2 further emphasizes the importance of vaccination and booster.

At a time when COVID-19 herd immunity has not yet been achieved, and we still need NPIs to fight the COVID-19 epidemic, so it is important to understand NPI priorities. The priority of NPI during the early stage of the COVID-19 pandemic in this study is consistent with previous studies (27). Restrictions in international and domestic travel make sense in preventing infection introduction (49, 50), especially given that travel has played a central role in the global spread of previous SARS epidemic (51). Cancelation of public events and gatherings are beneficial in reducing COVID-19 mortality and reproductive numbers, which have been shown in several studies (12, 28, 52). The strong impact of above NPI combinations on COVID-19 mortality may result from the fact that they are both mandatory policies and public facility closures which are easier to implement (53). The study found that “cancelation of public events and gatherings” have a greatest impact on COVID-19 mortality among the five common NPI combinations after vaccination rate reaching 70%. The prominent importance of “cancelation of public events and gatherings” to COVID-19 mortality has also been examined in previous studies (27, 54, 55). This NPI combination contributed to curb the spread of COVID-19 by preventing exposure to numerous and dense locations, where social distancing rules are more likely to be violated and contact tracing is difficult (55). In addition, there are studies demonstrating that the stricter implementation of “cancelation of public events and gatherings” will bring about a better suppression effect on the incidence and time-varying reproduction number of COVID-19 (52, 54). Perhaps COVID-19 public health experts can take this into account in the future to formulate more reasonable COVID-19 mitigation policies.

There are three limitations to this study. First, the intervention variable encoding of the Oxford COVID-19 Government Response Tracker relied on government announcements. However, the announcement did not guarantee effective policy implementation. Second, this research does not cover all mitigation policies that countries might apply (such as requirements for masks, hand hygiene, increased healthcare funding, ventilators, and protective equipment). Finally, many interventions were implemented simultaneously, making it difficult to completely isolate the effect of each other.

## Conclusion

Continued efforts are still needed to promote vaccination to build sufficient immunity to COVID-19 in the population. Until herd immunity is achieved, NPI is still important for COVID-19 prevention and control. At the beginning of the COVID-19 pandemic, the stringency of NPI implementation had a significant negative association with COVID-19 mortality; however, this association was no longer significant after the vaccination rate reached 70%. As vaccination progresses, “cancelation of public events and gatherings” become more important for COVID-19 mortality.

## Data availability statement

Publicly available datasets were analyzed in this study. This data can be found here: The datasets analyzed during the current study are available in the (Our World in Data) repository (https://ourworldindata.org/coronavirus).

## Author contributions

CY and HW contributed to conception and design of the study. HW organized the database, performed the statistical analysis, and wrote the first draft of the manuscript. CY, HW, CX, FS, YL, FW, XL, GQ, JB, QH, and RM revised the final manuscript. All authors contributed to manuscript revision, read, and approved the submitted version.
