# Supplementary material for: Non-pharmacological interventions of travel restrictions and cancelation of public events had a major reductive mortality affect during pre-vaccination coronavirus disease 2019 period
Source: Front Med (Lausanne). 2022 Aug 22;9:914732. doi: 10.3389/fmed.2022.914732 (PMC9441752; doi:10.3389/fmed.2022.914732)
Supplement: Supplementary file 1 [file Data_Sheet_1.pdf]

Supplementary Materials

This supplementary material presents the following in order: names of the 34 countries included in this study; definitions of the stringency of the 9 NPIs included in this study; baseline descriptions of the 34 countries included in this study; health-related covariates for the 34 countries included in the study; results of linear regression on OSI and COVID-19 Mortality; the performance of random forest model "RF1" and "RF2" in training and test set; Spearman coefficients of three importance rankings of NPI combinations in random forest model "RF1" and "RF2"; P-value of correlations of three importance rankings of NPI combinations in random forest model "RF1" and "RF2".

The 34 countries included in this study are: Argentina; Australia; Belgium; Brazil; Brunei; Canada; Chile; China; Costa Rica; Cuba; Denmark; Ecuador; Finland; France; Iceland; Ireland; Italy; Japan; Malaysia; Malta; Mauritius; Netherlands; New Zealand; Norway; Portugal; Qatar; Singapore; South Korea; Spain; Sri Lanka; Sweden; United Arab Emirates; United Kingdom; Uruguay.

Based on the codebook provided by the Oxford COVID-19 Government Response Tracker, Blavatnik School of Government, University of Oxford (1), we present the definition of the stringency of the nine NPIs included in this study as follows:

Supplementary Table 1 The definition of the stringency of the nine NPIs included in this study

	0	1	2	3	4
**school closures**	no measures	recommend closing or all schools open with alterations resulting in significant differences compared to non-Covid-19 operations	require closing (only some levels or categories, eg just high school, or just public schools)	require closing all levels	
**workplace closures**	no measures	recommend closing (or recommend work from home) or all businesses open with alterations resulting in significant differences compared to non-Covid-19 operation	require closing (or work from home) for some sectors or categories of workers	require closing (or work from home) for all-but-essential workplaces (eg grocery stores, doctors)	

**cancellation of public events**	no measures	recommend cancelling	require cancelling		
**restrictions on public gatherings**	no restrictions	restrictions on very large gatherings (the limit is above 1000 people)	restrictions on gatherings between 101-1000 people	restrictions on gatherings between 11-100 people	restrictions on gatherings of 10 people or less
**closures of public transport**	no measures	recommend closing (or significantly reduce volume/route/means of transport available)	require closing (or prohibit most citizens from using it)		
**stay-at-home requirements**	no measures	recommend not leaving house	require not leaving house with exceptions for daily	require not leaving house with minimal exceptions (eg allowed to leave once a	

			exercise, grocery shopping, and 'essential' trips	week, or only one person can leave at a time, etc)	
**restrictions on internal movements**	no measures	recommend not to travel between regions/cities	internal movement restrictions in place		
**international travel controls**	no restrictions	screening arrivals	quarantine arrivals from some or all regions	ban arrivals from some regions	ban on all regions or total border closure
**public information campaigns**	no Covid-19 public information campaign	public officials urging caution about Covid-19	coordinated public information campaign (eg across traditional and social media)		

Supplementary Table 2 Health-related covariates for the 34 countries included in the study

Location	Population density	Aged 65 older (%)	GDP per capita	Extreme poverty (%)	Diabetes prevalence (%)	Female smoker prevalence (%)	Male smoker prevalence (%)	Hospital beds per thousand	Life expectancy	Human development index
Argentina	16.177	11.198	18933.907	0.6	5.5	16.2	27.7	5	76.67	0.845
Australia	3.202	15.504	44648.71	0.5	5.07	13	16.5	3.84	83.44	0.944
Belgium	375.564	18.571	42658.576	0.2	4.29	25.1	31.4	5.64	81.63	0.931
Brazil	25.04	8.552	14103.452	3.4	8.11	10.1	17.9	2.2	75.88	0.765
Brunei	81.347	4.591	71809.251		12.79	2	30.9	2.7	75.86	0.838
Canada	4.037	16.984	44017.591	0.5	7.37	12	16.6	2.5	82.43	0.929
Chile	24.282	11.087	22767.037	1.3	8.46	34.2	41.5	2.11	80.18	0.851
China	147.674	10.641	15308.712	0.7	9.74	1.9	48.4	4.34	76.91	0.761

Costa Rica	96.079	9.468	15524.995	1.3	8.78	6.4	17.4	1.13	80.28	0.81
Cuba	110.408	14.738			8.27	17.1	53.3	5.2	78.8	0.783
Denmark	136.52	19.677	46682.515	0.2	6.41	19.3	18.8	2.5	80.9	0.94
Ecuador	66.939	7.104	10581.936	3.6	5.55	2	12.3	1.5	77.01	0.759
Finland	18.136	21.228	40585.721		5.76	18.3	22.6	3.28	81.91	0.938
France	122.578	19.718	38605.671		4.77	30.1	35.6	5.98	82.66	0.901
Iceland	3.404	14.431	46482.958	0.2	5.31	14.3	15.2	2.91	82.99	0.949
Ireland	69.874	13.928	67335.293	0.2	3.28	23	25.7	2.96	82.3	0.955
Italy	205.859	23.021	35220.084	2	4.78	19.8	27.8	3.18	83.51	0.892
Japan	347.778	27.049	39002.223		5.72	11.2	33.7	13.05	84.63	0.919
Malaysia	96.254	6.293	26808.164	0.1	16.74	1	42.4	1.9	76.16	0.81
Malta	1454.037	19.426	36513.323	0.2	8.83	20.9	30.2	4.485	82.53	0.895
Mauritius	622.962	10.945	20292.745	0.5	22.02	3.2	40.7	3.4	74.99	0.804

Netherlands	508.544	18.779	48472.545		5.29	24.4	27.3	3.32	82.28	0.944
New Zealand	18.206	15.322	36085.843		8.08	14.8	17.2	2.61	82.29	0.931
Norway	14.462	16.821	64800.057	0.2	5.31	19.6	20.7	3.6	82.4	0.957
Portugal	112.371	21.502	27936.896	0.5	9.85	16.3	30	3.39	82.05	0.864
Qatar	227.322	1.307	116935.6		16.52	0.8	26.9	1.2	80.23	0.848
Singapore	7915.731	12.922	85535.383		10.99	5.2	28.3	2.4	83.62	0.938
South Korea	527.967	13.914	35938.374	0.2	6.8	6.2	40.9	12.27	83.03	0.916
Spain	93.105	19.436	34272.36	1	7.17	27.4	31.4	2.97	83.56	0.904
Sri Lanka	341.955	10.069	11669.077	0.7	10.68	0.3	27	3.6	76.98	0.782
Sweden	24.718	19.985	46949.283	0.5	4.79	18.8	18.9	2.22	82.8	0.945
United Arab Emirates	112.442	1.144	67293.483		17.26	1.2	37.4	1.2	77.97	0.89

United Kingdom	272.898	18.517	39753.244	0.2	4.28	20	24.7	2.54	81.32	0.932
Uruguay	19.751	14.655	20551.409	0.1	6.93	14	19.9	2.8	77.91	0.817
**IQR**	237.113	8.547	23915.478	0.58	4.20	14.30	13.98	1.36	4.70	0.114

Note: Empty represent missing data, we use predictive mean matching method to fill it. IQR: interquartile range.

Supplementary Table 3 Baseline description for the 34 countries included in the study

Location	MRT date	MRT OSI	MRT mortality	VRT date	VRT OSI	VRT mortality
Argentina	2020/3/27	100.00	2.87	2021/10/21	42.59	3.01
Australia	2020/3/18	36.11	0.40	2021/10/15	74.54	2.84
Belgium	2020/3/11	19.44	83.79	2021/8/4	47.22	26.42
Brazil	2020/3/24	71.76	27.94	2021/9/27	54.17	11.41
Brunei	2020/3/28	52.78	0.68	2021/10/4	79.63	8.61
Canada	2020/3/18	61.11	23.06	2021/7/20	60.65	10.16
Chile	2020/3/26	73.15	29.61	2021/7/15	84.72	25.55
China	2020/2/2	77.31	0.32	2021/8/26	76.39	0.00
Costa Rica	2020/3/21	71.30	0.31	2021/10/18	54.63	9.65
Cuba	2020/3/28	66.67	0.76	2021/9/18	71.76	14.04
Denmark	2020/3/15	65.74	10.41	2021/7/25	47.22	12.47
Ecuador	2020/3/20	93.52	25.31	2021/10/28	57.87	4.04
Finland	2020/3/25	68.52	5.91	2021/8/22	38.43	9.91
France	2020/3/7	43.98	44.17	2021/8/19	66.67	15.70
Iceland	2020/3/21	53.70	2.91	2021/6/23	37.96	2.04
Ireland	2020/3/11	11.11	34.84	2021/8/11	44.44	17.12
Italy	2020/2/27	64.35	57.59	2021/8/28	55.56	13.83
Japan	2020/3/16	40.74	0.77	2021/9/29	50.46	0.62
Malaysia	2020/3/21	73.15	0.37	2021/9/23	64.81	20.77

Malta	2020/4/8	87.04	1.74	2021/7/2	49.07	10.85
Mauritius	2020/3/21	50.00	0.79	2021/10/21	32.41	7.46
Netherlands	2020/3/10	20.83	35.68	2021/7/24	41.67	18.45
New Zealand	2020/4/10	96.30	0.43	2021/10/19	81.02	0.45
Norway	2020/3/14	46.30	4.57	2021/8/24	38.89	9.04
Portugal	2020/3/19	82.41	15.50	2021/8/2	58.33	15.51
Qatar	2020/3/28	86.11	3.86	2021/7/31	50.00	0.58
Singapore	2020/3/21	36.11	0.48	2021/7/9	50.93	14.52
South Korea	2020/2/26	55.56	0.55	2021/9/17	47.22	6.30
Spain	2020/3/7	11.11	60.66	2021/8/4	47.69	16.17
Sri Lanka	2020/4/4	100.00	0.05	2021/10/26	68.52	6.16
Sweden	2020/3/13	30.56	52.49	2021/9/26	37.04	4.81
United Arab Emirates	2020/3/20	45.37	3.15	2021/7/5	60.65	3.25
United Kingdom	2020/3/14	12.96	59.29	2021/8/24	43.98	24.61
Uruguay	2020/3/28	62.96	0.77	2021/7/15	61.11	8.75

Abbreviation: MRT date, the date the COVID-19 mortality rate threshold was reached; MRT OSI, the OSI on the date of reaching the COVID-19 mortality rate threshold; MRT mortality, the cumulative death rate per 100,000 people on June 30, 2020; VRT date, the date the COVID-19 vaccination rate threshold was reached; VRT OSI, the OSI on the date of reaching the COVID-19 vaccination rate threshold; VRT mortality, the

cumulative death rate per 100,000 people between VRT date and December 31, 2021.

Supplementary Table 4 The association between OSI and COVID-19 mortality

**Lm1**					**Lm2**				
	Estimate	Std.Error	t value	*p*-value		Estimate	Std.Error	t value	*p*-value
(Intercept)	-17294.28	9763.40	-1.77	0.10	(Intercept)	165.10	1146.56	0.14	0.89
MRT OSI	-0.71	0.17	-4.24	<0.01	VRT OSI	0.06	0.18	0.35	0.73
Adjusted R-squared:		0.71			Adjusted R-squared:		0.06		
p-value:		<0.01			*p*-value:		0.58		

Note: MRT OSI, the OSI on the date of reaching the COVID-19 mortality rate threshold; VRT OSI, the OSI on the date of reaching the COVID-19 vaccination rate threshold; Std.Error: Standardized error.

Supplementary Table 5 The performance of random forest model "RF1" and "RF2" in training and test set

	MAE	MSE	RMSE	MAPE
RF1 in training set	4.70	43.23	6.57	0.44
RF1 in test set	16.37	389.60	19.74	1.49
RF2 in training set	2.48	9.40	3.07	0.32
RF2 in test set	4.21	22.92	4.79	0.40

Note: RMSE: root mean square error; MAE: mean absolute error; MSE: mean square error; MAPE: mean absolute percentage error.

Supplementary Table 6 Spearman coefficients of three importance rankings of NPI combinations in random forest model "RF1" and "RF2"

	RF1			RF2		
	NPI combinations ranking by permutation importance	NPI combinations ranking by Gini importance	NPI combinations ranking by SHAP importance	NPI combinations ranking by permutation importance	NPI combinations ranking by Gini importance	NPI combinations ranking by SHAP importance
NPI combinations ranking by permutation importance	1.00	0.70	0.70	1.00	0.60	0.60
NPI combinations ranking by Gini importance	0.70	1.00	0.70	0.60	1.00	1.00
NPI combinations ranking by SHAP importance	0.70	0.70	1.00	0.60	1.00	1.00

Note: SHAP: Shapley Additive Explanations; NPIs: non-pharmacological interventions.

Supplementary Table 7 *P*-value of correlations of three importance rankings of NPI combinations in random forest model "RF1" and "RF2"

	RF2			RF2		
	NPI combinations ranking by permutation importance	NPI combinations ranking by Gini importance	NPI combinations ranking by SHAP importance	NPI combinations ranking by permutation importance	NPI combinations ranking by Gini importance	NPI combinations ranking by SHAP importance
NPI combinations ranking by permutation importance		0.19	0.19		0.28	0.28
NPI combinations ranking by Gini importance	0.19		<0.01	0.28		<0.01
NPI combinations ranking by SHAP importance	0.19	<0.01		0.28	<0.01	

Note: SHAP: Shapley Additive Explanations; NPIs: non-pharmacological interventions.

Reference

1. the Oxford COVID-19 Government Response Tracker BSoG, University of Oxford. Codebook for the Oxford Covid-19 Government Response Tracker. 2021 2022/1/12. Available from: https://github.com/OxCGRT/covid-policy-tracker/blob/master/documentation/codebook.md.
